# Comparing private and public transport access to diabetic health services across inner, middle, and outer suburbs of Melbourne, Australia

**DOI:** 10.1186/s12913-018-3079-9

**Published:** 2018-04-13

**Authors:** Rebecca Madill, Hannah Badland, Suzanne Mavoa, Billie Giles-Corti

**Affiliations:** 1 Centre for Health Equity, School of Population and Global Health, The University of Melbourne, Level 5, 207 Bouverie St, Parkville, VIC 3052 Australia; 2 Healthy Liveable Cities Group, Centre for Urban Research, RMIT University, Building 15, Level 3, Room 12, Office 3, 124 La Trobe Street, Melbourne, VIC 3000 Australia; 3 Non Communicable Disease Unit, Melbourne School of Population and Global Health, The University of Melbourne, Rm 504, Level 5, 333 Exhibition Street, Melbourne, VIC 3010 Australia

**Keywords:** Health equity, Urban growth areas, GIS, Accessibility, Public transport, Private vehicle

## Abstract

**Background:**

Melbourne, Australia is experiencing rapid population growth, with much of this occurring in metropolitan outer suburban areas, also known as urban growth areas. Currently little is known about differences in travel times when using private and public transport to access primary and secondary services across Melbourne’s urban growth areas. Plan Melbourne Refresh, a recent strategic land use document has called for a 20 min city, which is where essential services including primary health care, can be accessed within a 20 min journey. Type 2 diabetes mellitus (T2DM) is a major chronic condition in Australia, with some of Melbourne’s growth areas having some of the highest prevalence across Australia. This study explores travel times to diabetic health care services for populations residing in inner, middle and outer suburbs of metropolitan Melbourne.

**Method:**

Geographic information systems (GIS) software were used to map the location of selected diabetic primary and secondary health care service providers across metropolitan inner, middle, outer established, outer urban growth and outer fringe areas of Melbourne. An origin-destination matrix was used to estimate travel distances from point of origin (using a total of approximately 50,000 synthetic residential addresses) to the closest type of each diabetic health care service provider (destinations) across Melbourne. ArcGIS was used to estimate travel times for private transport and public transport; comparisons were made by area.

**Results:**

Our study indicated increased travel times to diabetic health services for people living in Melbourne’s outer growth and outer fringe areas compared with the rest of Melbourne (inner, middle and outer established). Compared with those living in inner city areas, the median time spent travelling to diabetic services was between 2.46 and 23.24 min (private motor vehicle) and 12.01 and 43.15 min (public transport) longer for those living in outer suburban areas. Irrespective of travel mode used, results indicate that those living in inner and middle suburbs of Melbourne have shorter travel times to access diabetic health services, compared with those living in outer areas of Melbourne. Private motor vehicle travel times were approximately 4 to 5 times faster than public transport modes to access diabetic health services in all areas.

**Conclusion:**

Those living in new urban growth communities spend considerably more time travelling to access diabetic health services – particularly specialists - than those living in established areas across Melbourne.

## Background

### Health services in urban growth areas

Melbourne, in Victoria, Australia is experiencing rapid population growth with the city expected to reach approximately 8 million residents by the year 2051 [1]. Much of this population growth is occurring in low density outer suburban “urban growth areas” up to 50-100kms away from major city centres [1–3] as well as in infill areas across inner and middle suburbs of Melbourne [1]. As urban growth areas develop, they need infrastructure and services to meet the increasing demand of the growing population [4] and ensure equitable access to services [5]. Plan Melbourne, has called for a 20 min city, which is where essential services including primary health care services can be accessed within a 20 min journey [1]. Currently access to some essential primary health care services falls below this policy imperative (as outlined in this research), particularly for Melbourne’s outer urban growth areas.

Primary health care services, such as general practitioners (GPs) and pharmacists, serve the majority of health care needs for consumers across Melbourne [5]. However, evidence suggests that a higher density of these services exists within inner and middle suburbs of Melbourne [6], compared with outer suburbs and urban growth areas. Whilst, there is evidence of considerable inequities of access to health services in rural compared with city areas [3, 7], there is little published research about access to services in urban growth areas compared with established areas of cities. For example, access to health care services is dependent on adequate transport infrastructure to ensure those residing in outer urban growth areas are not disadvantaged.

This potential inequity of access to health services is of concern as a higher prevalence of vulnerable groups such as indigenous, young, elderly and culturally and linguistically diverse (CALD) populations, are located in Melbourne’s outer urban growth areas [2, 8, 9]. Therefore, those in most need may have poorer access to primary health care services. As such increasing access to primary health care services can aid in reducing inequities by providing services where they required most [10]. Importantly, better access to primary health care services has other benefits such as reducing admission rates for preventable causes of hospitalisation [11].

### Type 2 diabetes mellitus

This study focused on health services required for patients with type 2 diabetes mellitus (T2DM). T2DM represents over 85% of all diabetes cases in Australia [12]. Between 2011 and 2012 T2DM affected approximately 1.7 million Australians and almost 185,000 Victorians [13]. T2DM is increasing and is projected to affect approximately 3.5 million Australians by 2033 [13]. Additionally, the Australian Bureau of Statistics (ABS) contends that 24.7% of all diabetics live in the most disadvantaged areas across Australia [14]. The north and west region of Melbourne, a known urban growth area, not only has the highest prevalence of all types of diabetes compared with any other regions in metropolitan capitals across Australia [15], it is also comprised of vulnerable populations such as increasing numbers of elderly people [16], highlighting the need to deliver appropriate health care to those most at risk.

T2DM is a complex and useful condition to use to highlight issues of access to health services. A person who presents with T2DM is required to access a range of primary and secondary health care services on a regular basis. These health services include GPs, diabetes educators, dieticians, endocrinologists, podiatrists, pharmacists, optometrists or ophthalmologists, and psychological services [17]. This provides an indication as to the number of health services required to manage a complex chronic condition.

### Spatial accessibility to primary and secondary health care services

Penchansky and Thomas [19] denote five domains of health care access: availability, such as the number and type of health services [18, 19]; accommodation, encompassing aspects such as opening hours; affordability, which includes cost of health services to individuals and governments; acceptability, such as the cultural appropriateness of heath care facilities; and accessibility, including the relationship between the physical location of health services to individuals and travel time [19]. Spatial accessibility to health services is a domain of accessibility, focusing on geographical elements of how people access services in relation to their daily activities [20] and is a focus of this study.

Neutens [21] contends that policy makers and governments are becoming more perceptive to providing adequate, equitable and accessible health care and that decreasing spatial barriers, such as minimising travel time and distance, can increase health care utilisation. Cromely and McLafferty [20] argue that access is a function of distance decay, such that, the further the distance of a health care facility from someone’s primary place of access (usually their home), the less utilisation of that facility for the individual. This is of particular relevance to urban growth areas, as people are moving into communities where health facilities and public transport infrastructure are still being established. As a consequence, residents may have to travel outside their neighbourhood to access required health services [2]. This finding is supported by Hawthorne and Kwan [22]; they measured access using geographical distance and quality of care and found those living in suburban areas in Ohio, United States, had reduced access to health care compared with those who lived inner-city.

The present study focuses on spatial access to health services, as it is a sensitive marker of health care access equity [20] and is pertinent when examining access to health care infrastructure in urban growth areas. Primary and secondary health services across inner, middle, outer established, outer urban growth and outer fringe areas of Melbourne were chosen for investigation. Primary health care services are, for the most part, the initial point of contact to the health care system [5] and accordingly it was hypothesised that there would be reasonable access to primary providers. Primary health care services generally do not require a referral to a specialist and include services such as GPs, pharmacists, physiotherapists and dieticians [23]. As T2DM requires management from a range of health care providers, secondary health care services were also included to ascertain equity of health services in order to manage this complex and chronic condition [5]. Secondary health services often include specialist services, such as endocrinologists and diabetic educators, and therefore were likely not as abundant as primary health services [23].

Apart from spatial access, this study considered transport access to diabetic health services. It has been argued that decreasing patients’ travel time and having a range of transport options available to access health services are essential for increased health service equity [24, 25]. The mode of travel to access health services also requires attention as few studies consider health services access via public transport [26], with the majority defining ‘access’ through private transport modes [3, 26, 27]. One study conducted in East Anglia, England, by Lovett et al. [26] showed that for their sample population, a majority (67%) lived within a 5 min car trip to their nearest GP. They measured public transport access in terms of frequency of services, with 82% of the study population having access to at least four return bus trips per day to GPs. However, their study also found that those who had limited access to public transport, defined as having one or more return day time bus service, were more likely to live further away from town centres, and had to rely on private transport to access their GP [26].

As Martin et al. [28] purport, even in cities in the most developed countries not everyone has access to private transport. Moreover individuals who rely on public transport to access health services are often more disadvantaged (e.g. those who are older, younger, disabled, CALD, or have a lower socioeconomic status) [2, 5, 8]. Transport disadvantage has implications for access to health services particularly for residents in Melbourne’s urban growth areas and the mode of transport options they have available when accessing diabetic health services. In general, however, research into the mode of transport when accessing primary and secondary health services (including diabetic), specifically in growth areas, is severely lacking [2].

### Significance of the research

Global urbanisation is rapidly increasing with 1 billion more people living in urban areas in 2014 compared with 2000 [29]. As rapid urbanisation and population growth continues there is growing awareness of the impact the built environment has on health [30]. There is a link between the built environment and modern ‘epidemics’ of non-communicable diseases, such as T2DM, cardiovascular disease and certain cancers [31, 32]. It has been argued that such ‘epidemics’ are related to factors such as physical inactivity and obesity, which are perpetuated by increasingly low density outer suburban urban environments, where there is poor access to public transport and services, and inversely a higher reliance in car usage [31, 32].

Melbourne, Australia, is facing a number of development pressures including adapting to an increased and ageing population, being economically competitive and increasing social inequality [33]. For some, labour and housing markets have created opportunity, however it has further marginalised those more disadvantaged residents, particularly for this living in areas where there is insufficient access to public transport, employment, education and other essential services [33].

Low density neighbourhoods with poor access to transport, services and public open space [34], − attributes which characterise urban growth areas – have been associated with lower levels of physical activity and decreased access to healthy foods. These are pre-cursors to chronic diseases such as T2DM and cardiovascular disease [34]. Thus, it is increasingly recognised that Australian cities will face considerable pressure in the years ahead [4, 35]. If new urban growth areas are not adequately planned, one concern is that disadvantage and poor health will increase for those living in these new communities [2, 36]. Given that Melbourne is in a population growth period, strategic development and plans for urban growth corridors are being made for the next 30 to 40 years [37]. Planning for health care services, transport, road networks and housing now, will impact access to the social determinants of health for communities in many years to come.

Whilst studies have identified differences in health services accessibility between urban and rural settings [3, 38], few studies have considered differences in access to health services within a metropolitan area. Therefore this research contributes knowledge to the field through the examination of intra-city variations and health services access for a case study disease.

Additionally, few studies have considered access to both primary and secondary health care providers. However through the examination of diabetic health services, this study was able to do both. This is also true for transport, where few studies have considered access to health services for both private and public transport; however given the importance public transport can have for disadvantaged populations, particular for health services access, it was included in this study.

## Methods

### Aim

The aim of the study was to examine spatial access (i.e. travel times) to a range of diabetic health services across five areas of Melbourne via private and public transport.

### Methods outline

In order to estimate travel times from home to health facilities via private and public transport this study: 1) identified locations of relevant primary and secondary health care facilities; 2) created a synthetic sample of home addresses across Melbourne; and 3) used network analysis to estimate times from the synthetic home addresses to the health facilities. Detailed methods for each of these steps are described below.

### Study area

**Fig. 1:**
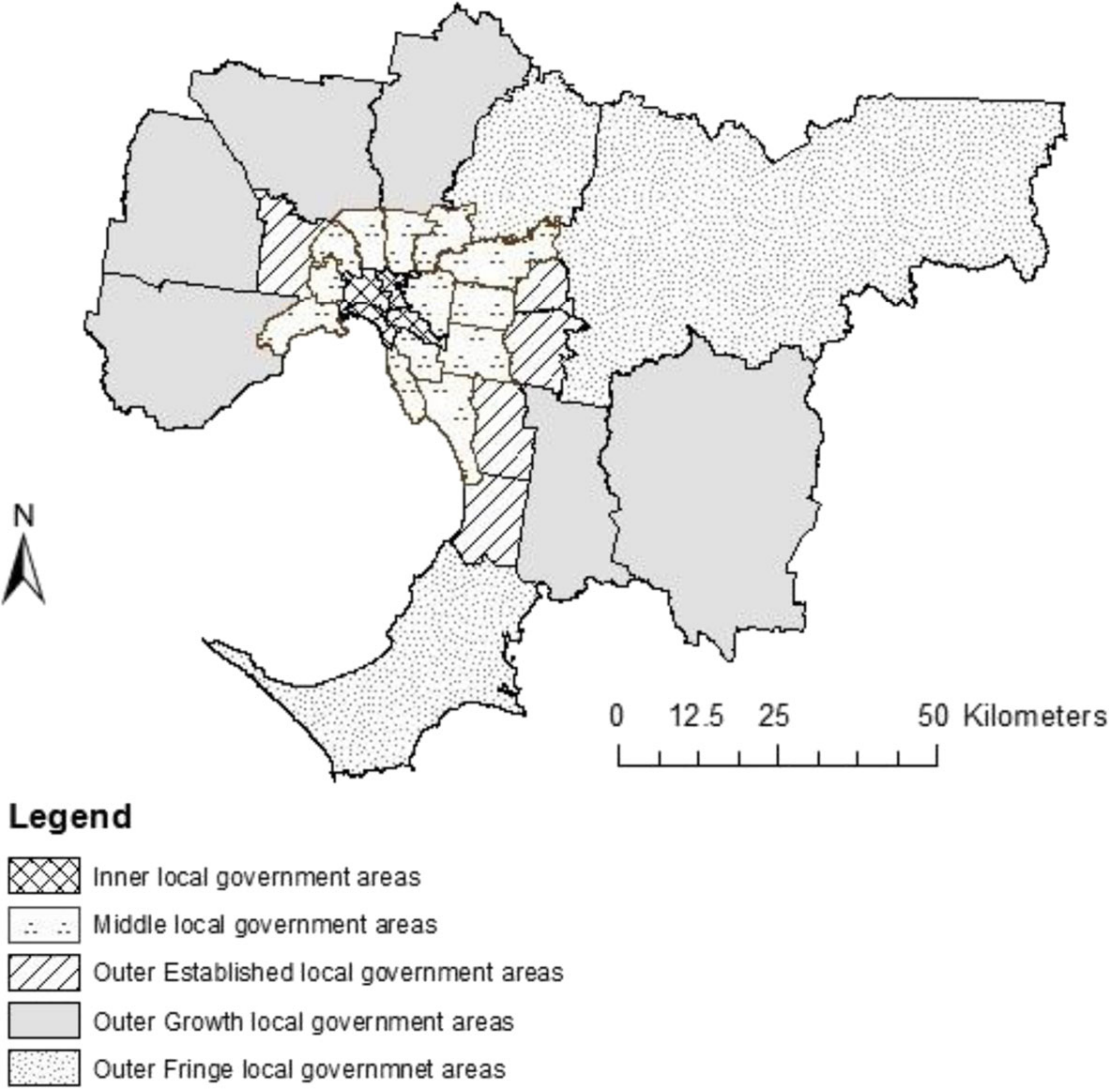
Metropolitan Melbourne. LGAs of Melbourne classified by inner, middle, outer established, urban growth areas and outer fringe areas. Figure 1 was created by author RM in the GIS software program ArcGIS

A Local Government Area (LGA) is a spatial unit for which a local council assumes responsibility of a geographical area [39]. As there are variations in population and infrastructure across outer metropolitan suburbs in Melbourne, this region was further divided into LGAs located in inner, middle, outer established, outer urban growth, and outer fringe areas (Fig. 1), as described below.

*Inner areas* were defined as areas of metropolitan Melbourne within approximately 7–10 km from the central business district [1]. *Middle areas* were defined as areas of metropolitan Melbourne within approximately 10–20 km from the central business district [1]. *Outer established areas* were defined as those on the outskirts of Melbourne that were not experiencing rapid population growth, and which have established infrastructure and suburbs [1]. *Outer urban growth areas* were defined as those located on the outer periphery of Melbourne, and classified as urban growth areas by the State Government of Victoria, as opposed to infill areas that existed in the inner and middle areas of Melbourne [1]. Outer urban growth areas were in a phase of rapid population growth requiring new services and infrastructure [1, 40]. *Outer fringe areas* were defined as those on the outskirts of Melbourne with increased development and population growth at the state average, but not experiencing the rapid population increase of urban growth areas [1, 40].

### Demographic data

Aggregated demographic data were sourced from the ABS 2011 Census Data [41] to provide some social context. ABS data indicates that percentage any car ownership is higher in Melbourne’s urban growth and fringe areas (Table 1). Additionally, percentage of people born in Australia was second lowest in urban growth areas and urban growth areas had residents with a younger median age.

**Table 1:** Demographic data for areas across Melbourne

Area of Melbourne	Median weekly household income in AU$ ± SD	Median age ± SD	% born in Australia ±SD	% household car ownership ±SD
Inner	1676.0 (171.1)	34.0 (3.3)	61.7 (10.8)	77.3 (11.0)
Middle	1321.0 (218.5)	38.0 (2.3)	62.3 (6.5)	88.8 (4.0)
Outer Established	1140.0 (175.9)	37.0 (1.5)	69.0 (16.8)	89.7 (3.0)
Urban Growth Area	1322.5 (73.0)	33.0 (0.9)	61.9 (7.1)	93.0 (1.1)
Outer Fringe	1281.0 (422.0)	39.0 (2.6)	79.5 (2.9)	93.9 (2.3)

Key: AU$ = Australian dollars; SD = Standard deviation

Source: Australian Bureau of Statistics (ABS) Census Data 2011 [41]

### Identifying diabetic health services

Diabetic health services were selected based on the services required for diabetic patients as recommended by Diabetes Australia Victoria [17]. These include diabetic educators, dieticians, endocrinologists, GPs, optometry, pharmacy, physiotherapists/exercise physiologist, podiatry and psychological services [17]. For the purpose of this study, primary health care providers were defined as services that did not routinely require a referral whereas secondary health services were defined as those services usually requiring referrals [23].

Melbourne is a large metropolitan area. Unlike some international cities Melbourne is a major capital with no neighbouring cities. It may have been the case that people accessed health care services in LGAs outside the study area and we collected health care service data in neighbouring areas of Melbourne to account for and minimise ‘edge effects’, that is, where people may access services close to home, yet lie outside that of metropolitan Melbourne’s boundary [20, 42].

### Health services data sources

GP, dietician, endocrinologist and diabetic educator addresses were sourced using the Victorian Health Services Directory [43]. After removing duplicate facilities, addresses were geocoded using geographic information systems (GIS). ArcGIS 10.2 (ESRI, Redlands, CA, USA) was used for all GIS analysis. Geocoded podiatry, optometry and pharmacy data were sourced from a commercial business points dataset [44] (Table 2). Due to lack of data psychological services were not included in this analysis.

**Table 2:** Health services: data source, date and number of health services collected

Type of health service	Health service	Data source	Data collection date	Number of services geo-coded
Primary	Dieticians	Victoria Health Services Directory	2015	*n* = 457 (Melbourne’s LGA’s and LGA’s adjacent to Melbourne)
Primary	GPs	Victoria Health Services Directory	2012	*n* = 1818 (across all of Victoria)
Primary	Optometry	Axiom business points from Pitney Bowes	2014	*n* = 853 (across all of Victoria)
Primary	Pharmacy	Axiom business points from Pitney Bowes	2014	*n* = 1930 (across all of Victoria)
Primary	Podiatry	Victoria Health Services Directory	2015	*N* = 221 (across all of Victoria)
Primary	Physiotherapy	Axiom business points from Pitney Bowes	2014	*n* = 919 (across all of Victoria)
Secondary	Diabetic educators	Australian Diabetes Education Association	2015	*n* = 213 (services included within a 200 km radius from Melbourne’s CBD)
Secondary	Endocrinologists	Victoria Health Services Directory	2015	*n* = 132 (Melbourne’s LGA’s and LGA’s adjacent to Melbourne)

### Residential data

Synthetic residential address points were created using address points sourced from Vicmap Planning 2013 [45]. A variety of planning zones exist across Melbourne [46]. As the aim of the study was to mimic people’s journey from their home to a health facility, all zoning classifications that could potentially have residential points were included in the data set. These included all residential zoned addresses as well as address points in the capital city zone, docklands zone, mixed land use zones and urban growth zone [46]. Residential addresses include any parcel lot where people can live (i.e. zoned as residential). Approximately 10,000 random address points were selected for each category of inner, middle, outer established, outer urban growth and outer fringe areas.

### Network analysis

Two origin destination (OD) matrix frameworks were created to estimate the travel time between each synthetic residential address and the nearest health care facility of each type for both private and public transport modes [47]. Time was used as a spatial measure of access to services, as it provides a more responsive measure of access to services than distance, while also accounting for access to transport modes [20]. Consumers are also more sensitive to travel time, rather than distance, when accessing and utilising health care services [20].

### Private motor vehicle network

The OD matrix was calculated for each trip, in minutes, using private transport from origin points (synthetic residential address data) to the destination point (geocoded health services). The private transport network was created using Vicmap Transport road centreline data [45].

Travel time varied for different roads at different times of day [48], therefore were calculated during off-peak day road times (between 10 am to 3 pm). Off peak travel times were used because of: a) the larger variation in travel conditions during peak times; and b) an assumption that many health services were more commonly accessed during non-peak periods. While this introduces bias for those who travel during peak periods, this is consistent across the entire road network. Any other potential single-time point disruptions that may occur along the road network (e.g. accidents, maintenance) were not accounted for in the calculations.

As detailed vehicle travel time data were unavailable for these origins and destinations, off-peak travel speeds for different types of roads in the road hierarchy were estimated (see Table 3) [49, 50]. Sign posted speed for each road type in the road hierarchy was sourced from the VicRoads traffic engineering manual [50] and VicRoads traffic monitor report, which provides average travel speeds by road category (e.g. freeway, arterial) and area (inner and outer). From this, average travel speeds were calculated (see Table 3). Average travel speeds were not available for local and collector roads. As shown in Table 3 the average travel speed for the arterial and sub-arterial networks across inner and outer regions were approximately half of the sign posted travel speed [48]. Therefore, we estimated the average travel speeds across the network for collector and local roads by using half the sign posted travel speeds, i.e. on a 50 km/h signed street, travel speeds were estimated to be 25 km/h.

**Table 3:** Road hierarchy and estimated private motor vehicle travel speeds

Road hierarchy type	Sign posted speed	Actual speed, off peak, inner LGA	Actual speed, off peak, middle LGA	Actual speed, off peak, outer established LGA	Actual speed, off peak, urban growth LGA	Actual speed, off peak, outer fringe LGA	Estimated private motor vehicle speed (i.e., Average, off peak, inner and outer)	Estimated private motor vehicle travel time
Freeway	100 km/h	70 km/h	70 km/h	80 km/h	80 km/h	80 km/h	75 km/h	20.83 m^− 1^
Arterial or Highway	80 km/h	32.5 km/h	32.5 km/h	43 km/h	43 km/h	43 km/h	37.75 km/h	10.49 m^− 1^
Sub-arterial	60 km/h	28 km/h	28 km/h	38 km/h	38 km/h	38 km/h	33 km/h	9.17 m^− 1^
Collector	50 km/h	Unknown	Unknown	Unknown	Unknown	Unknown	25 km/h	6.94 m^− 1^
Local	40 km/h	Unknown	Unknown	Unknown	Unknown	Unknown	20 km/h	5.56 m^−1^

Key: hr. = hour; km = kilometre; m^− 1^ = metres per second

Data source: VicRoads traffic monitor report [48] and VicRoads traffic engineering manual [50]

### Public transport network

Public transport stops, routes and timetables were sourced from Public Transport Victoria’s General Transit Feed Specification (GTFS) [51]. Public transport modes used in the analysis included trains, trams and bus services. A multi-modal network was created for the public transport network analysis using GTFS tools for ArcGIS [52]. The multi-modal network accounted for time spent walking to and/or travelling by public transport. Public transport and walking travel times were calculated from origin points (synthetic residential addresses) to the closest destination point (geocoded health services) and were estimated using the OD matrix. The OD matrix was calculated at the same day and time (12:00 pm, Wednesday).

### Statistical analysis

The final dataset contained shortest travel times in minutes from approximately 50,000 synthetic residential address points to eight diabetic health care services of interest, for both private and public transport in each of the five areas of Melbourne. Analysis of variance (ANOVA) testing was initially undertaken to determine significance of means between groups (i.e. inner, middle, outer established, outer growth and outer fringe), however due to the large sample investigated, the *p-values* were all significant. Therefore descriptive statistics were used to investigate differences across the five areas of Melbourne for each health service and between private and public transport.

## Results

**Fig. 2:**
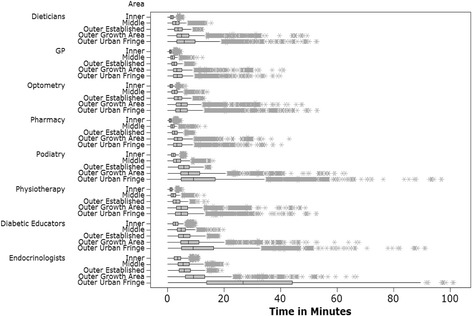
Private transport travel times. Travel times in minutes across areas of metropolitan Melbourne to diabetic health services via private transport. Figure 2 represents results from collected data and was created by author RM using the statistical software program Mini Tab

**Fig. 3:**
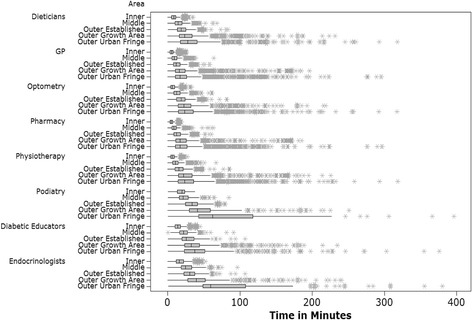
Public transport travel times. Travel times in minutes across areas of metropolitan Melbourne to diabetic health services via public transport. Figure 3 represents results from collected data and was created by author RM using the statistical software program Mini Tab

The findings for travel times to health services by private and public transport (Figs. 2 and 3) across the different areas are presented below. The results did indicate a number of outliers however the data were normally distributed. The results could have been truncated however the aim of the study was to understand the potential reality of travel times to access health services across Melbourne.

The results show travel time differences between private and public transport modes and primary and secondary health care services. Travelling to diabetic health services was between 5.89 (inner) to 4.02 (outer fringe) minutes faster by private motor vehicle when compared with public transport across all areas of Melbourne. Compared with those living in inner city areas, the median time spent travelling to diabetic services was between 2.46 and 23.24 min slower (private motor vehicle) and 12.01 and 43.15 min slower (public transport) for those living in outer suburban areas. Compared with middle suburbs travelling to diabetic services was between 1.1 min and 21.22 min slower (private motor vehicle) and 8.29 min and 40.62 min slower (public transport). Irrespective of travel mode used, results indicate that those living in inner and middle suburbs of Melbourne have faster travel times to access diabetic health services for both private and public travel modes, compared with those living in outer areas of Melbourne.

Road travel times were fastest when accessing primary health care services in inner Melbourne (GP median travel time in minutes: 0.89 (private transport), 5.1 (public transport); access pharmacies median travel time in minutes: 0.86 (private transport), 5.89 (public transport). Of note were the quick travel times when traveling to some GPs (0.89 min) and pharmacists (0.86 min). It was theorised that if a residential address was next to a GP or pharmacist, this would account for these travel times across the road network, as the analysis did not take into account time getting to the car, waiting in traffic, or parking. Travel times in Melbourne’s outer growth and fringe areas were similar for primary health care services. For example to access pharmacies, the median travel time in minutes was 3.51 (outer urban growth) and 3.26 (outer fringe) for private transport and 17.13 (outer urban growth) and 17.20 (outer fringe) for public transport. Time taken to access secondary health care services were slower compared with primary health care services. The slowest travel times were for specialist secondary health care services such as endocrinologists; median travel times for outer growth areas and outer fringe areas were 9.81 and 26.72 min respectively (private transport), and 39.3 and 59.25 min respectively (public transport).

## Discussion

### Access to diabetic health services in Melbourne’s growth areas

This research highlights that access to diabetic health services is faster in inner and middle areas of Melbourne compared with established, urban growth areas and fringe areas, irrespective of transport mode. Consistent with Lovett et al.’s [26] research, private transport was consistently shown to have faster travel times when accessing all health services compared with public transport modes. Our findings show that inner Melbourne had the fastest time spent travelling to access primary health care services (pharmacies and GPs). Those living in the outer urban growth and outer fringe areas of Melbourne experienced the slowest travel times when accessing specialist secondary health care services (endocrinologists).

Not surprisingly, residents living in Melbourne’s inner suburbs have the greatest spatial access to diabetic health services compared with other areas of Melbourne. For the most part, access declines linearly, whereby middle suburbs have greater access than outer established suburbs, and so forth. Our research confirms that access was even poorer in urban growth areas.

Our results also showed less variability in private motor vehicle and public transport travel times across inner and middle areas of Melbourne compared with outer suburban areas. This is in part due to the greater variability in the infrastructure available in outer suburbs across Melbourne. For example, in more established outer suburban areas more transport infrastructure and services were available in comparison with many of the new urban growth areas [1, 2]. Additionally, the Victorian Integrated Survey of Travel and Activity 2007–08 (VISTA 07–08) found that median distances to travel to work were higher in urban growth areas when compared with suburbs located closer to the city [53]. For example, the Melbourne metropolitan median travel distance to work was 14.3 km, yet for those living in Melton (one of Melbourne’s urban growth areas) the median distance was 32.5 km [53]. A study by Currie and Senbergs [54] considered households with ‘high car ownership on low incomes’ (HCOOLI) to explore car usage in Melbourne’s urban growth areas. Findings suggested that car usage by HCOOLI households in outer Melbourne was 5.2% more frequent, and car trips 38% longer than HCOOLI households in the middle suburbs of Melbourne [54]. Given that approximately 40% of Melbourne’s health services are located within a 10 km radius of the central business district [5], work travel times may serve to provide a crude indication that if health facilities continue to be located centrally in Melbourne, people living in urban growth areas need to travel further to access these services.

### Primary and secondary health care services

Not surprisingly the least time spent travelling to health services were for primary health care services. In regards to health services hierarchy, primary health care services are usually the first point of entry into the health care system and are likely to be accessed by many different populations, and most frequently [5]. Specialist services such as diabetic educators and endocrinologists serve particular sub-groups, for example those living with T2DM, and travel times to these services were the slowest across both modes of transport. Few studies have considered access to both primary and secondary health services in regards to travel times and travel mode, however it is generally accepted that travel times to primary health care services should be faster than secondary health care services. By way of example, Haynes et al. [55] found that 99% of their study population in East England could access a GP within a 17 min journey. Whereas, an American study by Onega et al. [56] considering access to specialised cancer services, found that median travel time to National Cancer Institute designated cancer centres was 78 min.

In our study, there was marked variability in travel times by transport mode by urban area typology when accessing both primary and secondary health care services; some residents in urban growth areas travelled up to 196 min via public transport to access a GP. This finding is inconsistent with policy directions in Melbourne. The state government’s recent strategic land use document ‘Plan Melbourne Refresh’ [1] has called for a 20 min city, where residents should be able to access essential services within a 20 min trip regardless of age or ability. These results suggest that whether this level of access is possible via private or public transport currently depends on the area of residence. Travel times of up to 196 min to reach basic primary health care services observed in this study, are clearly beyond this threshold and highlight significant health service access inequities in some areas within Melbourne. To improve access to health services in Melbourne’s outer urban growth areas, integrated planning is required: i.e., health planners providing more localised primary health care services – even if delivered in temporary locations in growth areas – and transport planners ensuring access to those services by providing accessible and frequent public transport choices [1].

This research supports that those living with TD2M in outer areas of Melbourne, will need to spend considerably more time travelling to access essential diabetic health services than those living closer to the city. While not inordinate travel times for primary care services, it appears that in order to achieve equitable access, specialist services may require an alternative delivery model to provide for the increasing number of those living in urban growth areas and fringe areas of cities.

### Private versus public transport

Residents living in Melbourne’s urban growth areas have been shown to experience transport disadvantage, as they live in car-dependant areas with poor access to public transport [54, 57, 58]. This was confirmed by our study. Additionally, urban growth areas in Melbourne are known to have a diverse population with residents from a range of backgrounds such as CALD groups, youth populations and the elderly [5, 59]. This finding is comparable with a study conducted by Roeger et al. [60] which considered spatial access to GPs in metropolitan Adelaide, Australia and concluded those most disadvantaged and requiring access to primary health care services were those living in outer suburbs and with a lower socio economic status.

People living in growth areas are faced with a complex transport situation: the remoteness coupled with a lack of other transport mode options fosters reliance on private car ownership [2, 40]. Currie et al. [54, 57] have explored factors influencing transport disadvantaged in Melbourne’s outer urban growth areas, including the forced transport costs often related to car ownership, due to poor access to other transport options. The financial cost of travelling to health services can be prohibitive for disadvantaged populations, especially those who rely on private transport, particularly hired transport, such as taxis [61].

This has implications when accessing health services, particularly for vulnerable populations who have a greater reliance on public transport to access health services [57, 59]. Residents in Melbourne’s growth areas face a ‘triple disadvantage’ situation; they have less access to health care services and public transport to get to services, while a greater proportion of them are more vulnerable [2, 5, 59].

#### Limitations

Spatial access of health service locations offers one dimension of health services access. Other factors that could have been considered as part of access include cultural appropriateness [18, 62], opening hours [18, 19], cost [19, 20], and how people access health services in respect to their daily activity patterns (e.g. services around the workplace) [20, 63]. Additionally an assumption was made that people were accessing the closest type of destination, whereas they likely have a preferred provider who might not be the closest one.

Also not all diabetic health services were analysed. For example, psychological services were not included. Psychological services provide a valuable health service for diabetic patients as well as the community at large [17]. Additionally, the health service data sets accessed from a private provider may have been incomplete or had some inaccuracies. For example, locations were provided for private podiatry clinics, but did not include other locations where podiatrists may practise, such as community health centres.

There were some limitations to the road network analysis. The road network was based on a formula using the travel speeds for each road type (e.g. freeway, arterial roads) and distance to the nearest of each type of health service. Whilst some concession was made to adjust for the average travel speed along the road network, it did not take into account other congestion variations, such as road works, accidents, parking, traffic, lights, intersections and peak travel times. Also, the model did not simulate a situation where someone may have driven to a train station and then used public transport. In Melbourne approximately 61% walk to train station, approximately 23% catch a bus or tram to a train station and approximately 12% drive their car to a train station [64–66].

Furthermore, this study lacks health outcomes, health utilization and socio-economic status data. Though the study included limited demographic data it was beyond its scope to analyse demographic and social data in relation to travel time access across areas of Melbourne. However, this could be pursued in further studies. Also, the residential addresses in this study were a sample of where residents potentially live; the study did not consider someone who actually had diabetes and their true exposure, or their unique need to utilise health care services. Instead the research was based on synthetic residential address data to estimate journey times. The actual experience of accessibility to a high demand service might provide additional contextual information. We were unable to test this using synthetic data. All health services were weighted equally, but it is likely that some services are more important than others depending on the progression of diabetes, and this was unable to be tested through this study design. There is also a danger of the ecological fallacy, whereby inferences are made about individuals based on aggregated data.

Additionally, for someone who has stable T2DM they may require review from their GP every 3–12 months, dependant on other co-morbidities; however someone who has increased risk of complications may require monitoring from their GP every 6–12 weeks [67]. One method to improve access to diabetes health services includes the use of eHealth which can include mHealth (mobile health), telehealth, electronic health records, eLearning, social media and very large data sets known as big data [68]. This present study did not test the efficacy of eHealth into diabetic health services access as it was outside the scope of the current research, however there are number of Australian studies that have investigated the impacts of ehealth delivery [69–72] and it is an area for further research when considering diabetic health services access in urban growth areas.

## Conclusion

Melbourne, Victoria, is currently experiencing a rapid population increase, specifically in urban growth areas and this study identifies considerable time differences in accessing diabetic health care services, particularly specialist services. Urban growth areas are often considered attractive places to live because of their perceived affordability, however as many disadvantaged populations live within these areas health services should be planned to meet current needs and to minimise any potential health services access inequities.

Understanding the current situation of diabetic health service access in urban growth areas will help health and urban planners respond by providing health services accordingly. Plan Melbourne has called for a 20 min city where essential services such as primary health care should be able to be accessed within a 20 min journey of people’s homes. So far, this has not been achieved in growth areas; however it provides a planning metric for which to aim for health services accessibility.

Planning for health services in Melbourne’s urban growth areas requires a co-ordinated approach from all sectors of government such as health, urban planning, transport and land use. Through integrated planning, providing health services closer to people’s homes will reduce travel times and increase equity of access for those who rely on public transport. As the population and demographics in urban growth areas continues to expand and change, further investigation is warranted to explore alternative ways to delivery diabetic health services to people living in these areas.

## Abbreviations

ABS: Australian Bureau of Statistics
ANOVA: Analysis of variance
CALD: culturally and linguistically diverse
GIS: Geographic Information Systems
GP: General Practitioner
GTFS: General Transit Feed Specification
HCOOLI: High car ownership on low incomes
hr.: Hour
km: Kilometre
LGA: Local Government Area
m^− 1^: Metres per second
T2DM: Type 2 Diabetes Mellitus
VISTA: Victorian Integrated Survey of Travel and Activity
